# Therapeutic Notes

**Published:** 1896-01-18

**Authors:** 


### THERAPEUTIC NOTES.

### BROMHYDRATE OF ARECOLINE.

Arecoline has been brought forward both for its aperient action and as a new myotic alkaloid. Mouquet[1] suggests arecoline, one of the alkaloids of areca nut, as a substitute for sulphate of eserine and pilocarpine, which are employed by veterinary surgeons for intestinal indigestion in horses. He found by experiments on horses that bromhydrate of arecoline produced salivation, diarrhœa, expulsion of flatus, and that it combined the actions of pilocarpin and eserine in these respects. Lavagna[2] finds that the hydrobromate contracts the pupil when applied locally. If a few drops of a 1 per cent. solution are put into the eye, there is first a feeling of warmth, with some spasm of the lid; tears flow, and there is some evidence of irritation, lasting about a minute, and followed by some hyperæmia of the conjunctiva and slight superficial injection of the cornea, which disappears in a few minutes. In about two minutes strong clonic contractions of the iris are seen, with decreased size of the pupil. The myosis is well marked in five minutes, and reaches its maximum in ten minutes. After a time slight dilatation is noticed. Some degree of megalopsia occurs in thirty to thirty-five minutes after the drug has been applied. Even after several applications nothing like headache is produced. Lavagna points out that arecoline acts on the ciliary muscle before it acts on the iris. The special feature in the action of arecoline is that it produces a very marked myosis, which lasts only a short time.

[1] Les Nouv. Rem., Nov. 24, 1894. [2] Therap. Monat, VII., 1895; Med. Chron., Oct., 1895.

